# Immunity against Delta and Omicron variants elicited by homologous inactivated vaccine booster in kidney transplant recipients

**DOI:** 10.3389/fimmu.2022.1042784

**Published:** 2023-01-09

**Authors:** Lei Zhang, Jiaqing Yang, Changchun Lai, Li Wan, Shilong Xiong, Weiya Kong, Zijian Liu, Pei Yu, Mingxiao Chen, Weikang Mai, Shahzad Akbar Khan, Min Deng, Lu Chen, Yu Lei, Qiang Zhou, Nan Yu, Pingchao Li, Zheng Chen, Tianxing Ji

**Affiliations:** ^1^ Kidney Transplant Department, The Second Affiliated Hospital of Guangzhou Medical University, Guangzhou, China; ^2^ State Key Laboratory of Respiratory Disease, Guangzhou Institute of Respiratory Health, The First Affiliated Hospital of Guangzhou Medical University, Guangzhou, China; ^3^ Provincial Key Laboratory of Immune Regulation and Immunotherapy, Department of Medical Laboratory, School of Laboratory Medicine and Biotechnology, Southern Medical University, Guangzhou, Guangdong, China; ^4^ Clinical Laboratory Medicine Department, Maoming People’s Hospital, Maoming, Guangdong, China; ^5^ Clinical Laboratory Medicine Department, The Second Affiliated Hospital of Guangzhou Medical University, Guangzhou, China; ^6^ State Key Laboratories of Respiratory Diseases, Guangdong-Hong Kong-Macao Joint Laboratory of Infectious Respiratory Disease, Guangzhou Institutes of Biomedicine and Health, Chinese Academy of Sciences, Guangzhou, China; ^7^ Laboratory of Pathology, Department of Pathobiology, University of Poonch Rawalakot, Azad Kashmir, Pakistan

**Keywords:** inactivated vaccine booster, binding antibody, cellular immunity, Omicron variants, Delta variants, kidney transplant recipients

## Abstract

**Background:**

A third mRNA vaccine booster is recommended to improve immunity against SARS-CoV-2 in kidney transplant recipients (KTRs). However, the immunity against SARS-CoV-2 Ancestral strain and Delta and Omicron variants elicited by the third dose of inactivated booster vaccine in KTRs remains unknown.

**Methods:**

The blood parameters related to blood cells count, hepatic function, kidney function, heart injury and immunity were explored clinically from laboratory examinations. SARS-CoV-2 specific antibody IgG titer was detected using an enzyme-linked immunosorbent assay. Cellular immunity was analyzed using interferon-γ enzyme-linked immunospot assay.

**Results:**

The results showed that there were no severe adverse effects and apparent changes of clinical laboratory biomarkers in KTRs and healthy volunteers (HVs) after homologous inactivated vaccine booster. A third dose of inactivated vaccine booster significantly increased anti-Ancestral-spike-trimer-IgG and anti-Ancestral-receptor binding domain (RBD)-IgG titers in KTRs and HVs compared with the second vaccination. However, the anti-Delta-RBD-IgG and anti-Omicron-RBD-IgG titers were significantly lower than anti-Ancestral-RBD-IgG titer in KTRs and HVs after the third dose. Notably, only 25.6% (10/39) and 10.3% (4/39) of KTRs had seropositivity for anti-Delta-RBD-IgG and anti-Omicron-RBD-IgG after booster, which were significantly lower than HVs (anti-Delta-RBD-IgG: 100%, anti-Omicron-RBD-IgG: 77.8%). Ancestral strain nucleocapsid protein and spike specific T cell frequency after booster was not significantly increased in KTRs compared with the second dose, significantly lower than that in HVs. Moreover, 33.3% (12/36), 14.3% (3/21) and 14.3% (3/21) of KTRs were positive for the Ancestral strain and Delta and Omicron spike-specific T cells, which were significantly lower than HVs (Ancestral: 80.8%, Delta: 53.8%, and Omicron: 57.7%).

**Conclusions:**

A third dose of inactivated booster vaccine may significantly increase humoral immunity against the Ancestral strain in KTRs, while humoral and cellular immunity against the Delta and Omicron variants were still poor in KTRs.

## Introduction

Severe Acute Respiratory Syndrome Coronavirus 2 (SARS-CoV-2) is continuously able to escape the protective immunity produced by natural infection or vaccination amid the ongoing Coronavirus Disease 2019 (COVID-19) pandemic. Recent studies showed that the predominant circulating strain of Delta and Omicron variants have much higher mutation sites than the Ancestral isolate, which greatly increases the difficulties in controlling the COVID-19 pandemic (1, 2). The fifteen mutations in the receptor binding domain of spike protein in Omicron variants confer greater immune escape against neutralizing antibodies (NAbs) elicited by vaccination or infection and enhanced contagiousness (2, 3). Accordingly, real word analysis demonstrated that vaccine efficacy against Omicron variants infection was significantly decreased after two doses of mRNA vaccination (4), which has increased concerns about the effectiveness of vaccination strategies in immunocompromised patients.

Kidney transplant recipients (KTRs) routinely administered with immunosuppressive drugs have higher risks of severe or prolonged SARS-CoV-2 infection, which could be fatal (5). Therefore, SARS-CoV-2 vaccination in KTRs is recommended (6). However, the immunogenicity of KTRs towards all the approved SARS-CoV-2 vaccine administration schemes, including mRNA vaccine, adenovirus vector vaccine and inactivated vaccine, is poor (7). Additionally, it was shown that the two-dose vaccination scheme in KTRs was associated with a 12.8- and 8.1-fold risk for COVID-19 hospital admission and death compared with healthy individuals (8). Further, although accumulating data suggest that a third homologous or heterologous booster could effectively improve the immune response against Ancestral strain in the general population (9), the seroconversion rate of NAbs against Delta and Omicron variants after the third dose of mRNA vaccine is still reported to be poor in KTRs (10–12). Currently, inactivated vaccine accounts for the highest number (45%) of the delivered dose of approved SARS-CoV-2 vaccines worldwide (13). However, there is no available data on immunity against the Ancestral strain and the Delta and Omicron variants elicited by homologous inactivated vaccine booster in KTRs.

## Material and methods

### Human participants and related study cohort

This observational study was conducted in accordance with the Declaration of Helsinki and was approved by the Ethics Committee of the Second Affiliated Hospital of Guangzhou Medical University (Approval No. 2021-hs-43). KTRs and healthy volunteers (HVs) were enrolled at the Second Affiliated Hospital of Guangzhou Medical University from June 20, 2021, to January 25, 2022. In total, 39 KTRs enrolled in previous study of two doses of inactivated vaccine scheme (14) and 39 HVs agreed to participate for the third homologous booster. After obtaining written informed consent, these participants were vaccinated with inactivated vaccine six months after completing two doses of inactivated vaccination. Further, the data of 40 KTRs and 23 HVs from a previous study on the immunogenicity of a two-dose inactivated vaccine program were included to evaluate the immunity response between two doses of inactivated vaccine and the third homologous booster (14). It should be mentioned 23 HVs cohort of the second dose is different from 39 HVs cohort of the third dose. Clinical parameters including age, sex, vaccination history and adverse effects (AE) were recorded after the third homologous booster. Inactivated SARS-CoV-2 vaccine administration can induce antibodies against all viral proteins, including anti-nucleocapsid protein (NP) antibodies, posing great challenges in the serological discrimination of vaccine response from infection (15, 16). SARS-CoV-2 RNA positive history was used to exclude infection history in this study because continuous detection of SARS-CoV-2 RNA in the general population and strict epidemic prevention and control measures have been executed in China. Based on this criterion, participants with a SARS-CoV-2 infection history were excluded.

### Sample collection and processing

Whole blood was collected from all enrolled participants at an average of 21 days post-second dose of inactivated vaccine (14) and 14 and 28 days post-booster (DPB). After centrifugation at 3000 rpm for 15 min, the plasma was separated for anti-SARS-CoV-2 antibody detection. Additionally, an equal amount of Roswell Park Memorial Institute (RPMI) 1640 culture medium (Gibco, USA) was added and thoroughly mixed. Peripheral blood mononuclear cells (PBMCs) were isolated from whole blood samples using the Lymphoprep™ density gradient medium (Alere Tech, USA) for T cell immune response analysis. 36 samples from KTRs at 14 DPB, 35 samples from KTRs at 28 DPB, and 26 samples from HVs after 14 and 28 DPB were available for Ancestral spike and NP-specific T cells evaluation. Of them, Delta and Omicron spike-specific T cell responses were evaluated for 13 samples from KTRs at 14 DPB, 21 samples from KTRs at 28 DPB, 14 samples from HVs 14 DPB, and 26 samples from HVs 28 DPB.

### Clinical laboratory tests

The clinical data related to blood cells count (white blood cell, lymphocyte, neutrophil, eosinophil, basophil, platelet, reticulocyte, low fluorescence reticulocyte, median fluorescence reticulocyte, high fluorescence reticulocyte, and hemoglobin), hepatic function biomarkers (alanine aminotransferase, total bilirubin, unconjugated and conjugated bilirubin, γ-glutamyl transferase, and alkaline phosphatase), kidney function biomarkers (serum creatinine, urea nitrogen, uric acid, Cystatin C, urine protein, urine microalbumin, and urine β2-microglobulin), heart injury biomarkers (creatine kinase, creatine kinase isoenzyme, lactate dehydrogenase, and aspartate aminotransferase) and immunity-related biomarkers (complement C3, C4, IgG, IgA, and IgM) were clinically detected from laboratory examinations before and 7, 14 and 28 days after the third booster.

### Antibody titers against SARS-CoV-2 detection

Antibody titers were determined using an enzyme-linked immunosorbent assay (ELISA) as previously reported (17). Briefly, 96-well ELISA plates were coated with proper concentrations of recombinant viral antigens including SARS-CoV-2 ancestral strain-spike trimer (Dongkang Biotech, China, 50 ng/well), ancestral strain-NP (Dongkang Biotech, China, 100 ng/well), ancestral strain-RBD (Dongkang Biotech, China, 180 ng/well), Delta variants-RBD (Fapon Biotech, China, 120 ng/well), and Omicron variants-RBD (Fapon Biotech, China, 180 ng/well) overnight and blocked by adding 100 μL of 5% milk blocking solution in each well of ELISA plate. Three folds of serially diluted serum samples starting from 1:20 were added to each plate well and incubated at 37 ℃ for 1 hour. After washing, each plate well was coated with 100 μL diluted anti-human IgG antibody conjugated with HRP (1:10000, Southern Biotech, USA) and incubated for 1 hour. Consequently, 50μL TMB substrate (Neobioscience, China) was added to the wells after washing five times. Lastly, the optical density (OD) value was measured at 450nm using a microplate absorbance reader (Tecan Sunrise, Switzerland). Antibody endpoint titers were determined according to the highest dilution, which gave an OD value higher than the mean + 3SD OD values of 3 serum pools from 45 already stored serums at the same dilution collected from healthy individuals in the year 2016 (17).

### Surrogate virus neutralization assay

The anti-SARS-CoV-2 NAbs ELISA Kit (Vazyme Biotech, China) was used qualitatively to detect RBD-angiotensin-converting enzyme2 (ACE2) interaction-blocking antibodies. Briefly, 80 μL of horseradish peroxidase (HRP)-conjugated RBD solution was added in a 96-well dilution plate with 8 μL plasma and 72 μL sample dilution buffer, and incubated at 37°C for 30 min. Then, 100 μL of this plasma/HRP-conjugated RBD mixture was transferred to a microplate coated with ACE2 and incubated at 37°C for 20 min. Then 100 μL TMB substrate solution was added after washing with diluted washing buffer, and incubated at room temperature for 15 min. The reaction was stopped with 50 μL of stop solution. Finally, the absorbance at 450 nm was obtained using a microplate absorbance reader (Tecan Sunrise, Switzerland). The inhibition rate was calculated by the following formula: inhibition rate = (1‒ absorbance of sample/mean absorbance of negative controls) × 100%. Anti-SARS-CoV-2 NAbs positivity was defined by an inhibition rate higher than or equal to 20% according to the manufacturer’s instructions.

### Detection of SARS-CoV-2 specific T cells frequencies

SARS-CoV-2 (including Ancestral strain, Delta variants, and Omicron variants) spike and ancestral strain NP specific T lymphocytes were detected using interferon-γ (IFNγ) enzyme-linked immunospot (ELISPOT) assay as previously described (14). Briefly, 2×10^5^ fresh PBMCs were added in each well of an anti-IFNγ pre-coated ELISPOT plate (Dakewe Biotech, China) and co-cultured with 50ng overlapping peptide pools of each SARS-CoV-2 variants spike including Ancestral strain, Delta variants, and Omicron variants (Genscript Biotech Corporation, China) or ancestral strain NP (Genscript Biotech Corporation, China) for 24 h, with dimethyl sulfoxide (Sigma, USA) as a negative control (NC). For positive control, 2×10^4^ PBMCs were stimulated with staphylococcal enterotoxin B (1 µg/mL, Merck, Germany). After completion of the chromogenic reaction, the spots were counted using the ImmunoSpot^®^ S6 UV Analyzer (Cellular Technology Limited, USA). The spot forming units (SFU) of each well was determined by subtracting spots of the unstimulated wells from the peptide-stimulated wells. The SFU of each sample was calculated using the means of duplicate wells and expressed as SFU/10^6^ PBMCs. The threshold for cellular immunity positivity was calculated as the mean + 3 SD SFU/10^6^ PBMC of unvaccinated, infection-naïve healthy donors (n=30) and KTRs (n=38) (18). This resulted in cut-off values for NP (ancestral strain) and spike (ancestral strain) specific positivity of 78.36 and 69.09 SFU/10^6^ PBMC respectively. In addition, the cut-off values of ancestral strain specific T cells were used to determine the positivity of Delta and Omicron variant spike T cells response in this study due to unavailable fresh PBMCs from unvaccinated, infection-naïve healthy donors for determining their positive threshold and high conserved spike T cell epitope between ancestral strain and Omicron variant (19).

### Statistical analysis

All statistical analyses were performed using the IBM SPSS Statistics software (version 22.0) or GraphPad Prism (version 7.0). Binding antibody titers were expressed as geometric mean titers (GMTs) and 95% confidence interval (CI). The mean (standard deviation [SD]) or median (interquartile range [IQR]) was used to present the continuous variables. Categorical variables are described as counts and percentages. The differences in proportions between two groups were determined using the Pearson Chi-square test. The independent group t-test (normal distribution) and Mann-Whitney U test (non-normal distribution) were used to compare continuous variables between groups. Paired data were analyzed using the Wilcoxon test. Spearman correlation analysis was used to explore the correlation between SARS-CoV-2 specific cellular and humoral immunity. A two-sided p-value ˂ 0.05 was considered statistically significant.

## Results

### Demographic characteristics and AE in KTRs and HVs after third homologous booster

A total of 39 KTRs and 39 HVs who completed two doses of inactivated vaccination were enrolled to evaluate immunogenicity by adding a third booster during the study (**Table 1**). The median age, sex and vaccine brand of the third dose of KTRs were similar to HVs. In addition, no significant difference was observed in terms of AE between KTRs and HVs after the booster (**Table 1**). Most KTRs (30/39,76.9%) and HVs (34/39, 87.1%) had no abnormalities after the third dose, except the feeling of pain at the injection site and fatigue, indicating that all AEs to the vaccine were mild, transient and self-limiting in KTRs and HVs after the booster. Regarding KTRs, the median interval between kidney transplant and first inoculation of inactivated vaccine was 49 months (IQR=60) (**Table 1**). Other related data of KTRs, including graft type, induction agent used and immunosuppressive drug administration, are shown in **Table 1**.

**Table 1:** The baseline characteristics and adverse events of enrolled healthy volunteers and kidney transplant recipients.

	HVs (n=39)	KTRs (n=39)	*p*-value
Age (median, IQR)	38 (15)	44 (16)	0.074
Female/Male (%)	11(28.2)/28(71.7)	8(20.5)/31(79.5)	0.429
Inactivated Vaccine brand of the third dose			
Sinopharm BIBP n (%)	9(23.1)	7(17.9)	0.575
CoronaVac n (%)	30(76.9)	32(82.1)	
Adverse effects after third dose			
No abnormalities n (%)	34(87.1)	30(76.9)	0.259
Pain at injection site n (%)	4(10.3)	2(5.1)	
Fatigue n (%)	0(0.0)	3(7.7)	
Dizziness n (%)	0(0.0)	1(2.6)	
Backache n (%)	0(0.0)	1(2.6)	
Tachycardia n (%)	0(0.0)	1(2.6)	
Injection site cyanosis n (%)	0(0.0)	1(2.6)	
Rhinorrhea n (%)	1(2.7)	0(0.0)	
Time since kidney transplant (months), median (IQR)		49 (60)	
Type of graft			
Kidney transplant (%)		37(95.0)	
Simultaneous pancreas-kidney transplant (%)		1(2.5)	
Simultaneous liver-kidney transplant (%)		1(2.5)	
Induction agent used			
ATG (%)		28(72.5)	
Basiliximab+ ATG (%)		7(17.5)	
Rituximab+ATG (%)		3(7.5)	
Cyclophosphamide (%)		1(2.5)	
Immunosuppression			
Tacrolimus+MMF+ Prednisone		37(95.0)	
Tacrolimus+Mizoribine+ Prednisone		1(2.5)	
Tacrolimus+MMF+Rapamycin+ Prednisone		1(2.5)	

KTRs, kidney transplants recipients; HVs, healthy volunteers; IQR, Inter quartile range; ATG, Anti-Thymocyte Globulin; MMF, mycophenolate mofetil.

### Dynamic of clinical laboratory routine biomarkers in KTRs and HVs after third homologous booster

Previous data showed that inoculation of inactivated vaccine might lead to elevated levels of renal function biomarker creatinine and a decrease in CD8+ T cells (20), which are of great importance for KTRs. However, our longitudinal analysis did not find significant alterations in the hematology, heart, kidney and hepatic function biomarkers in HVs and KTRs after booster (**Supplemental Tables 1**, **2**). Unexpectedly, reticulocyte counts in HVs were significantly lower than in KTRs at pre-booster, which were significantly increased at 14 ± 2 days after the third booster (**Figure S1A**). However, there was no significant alteration in reticulocyte counts in KTRs after booster compared to pre-booster (**Figure S1A**). Interestingly, compared to pre-booster, the immature reticulocyte fraction (IRF) was significantly increased in HVs at 7 ± 1 and 14 ± 2 DPB but then returned to pre-vaccinated levels at 32 ± 4 DPB (**Figure S1B**). Accordingly, the low fluorescence reticulocyte ratio was significantly decreased in HVs at 7 ± 1 and 14 ± 2 DPB, which then returned to pre-vaccinated levels at 32 ± 4 DPB (**Figure S1C**). However, similar observations were not observed in KTRs (**Figure S1C**). Serum conjugated bilirubin levels in KTRs decreased significantly at 7 ± 1, 14 ± 2 and 32 ± 4 DPB relative to pre-third vaccination (**Figure S1D**). Similarly, the third booster was associated with a transient decrease in conjugated bilirubin in HVs at 7 ± 1 DPB, which then returned to pre-vaccinated levels at 14 ± 2 and 32 ± 4 DPB (**Figure S1D**).

### SARS-CoV-2 specific antibody in KTRs and HVs after homologous booster

**Figure 1:**
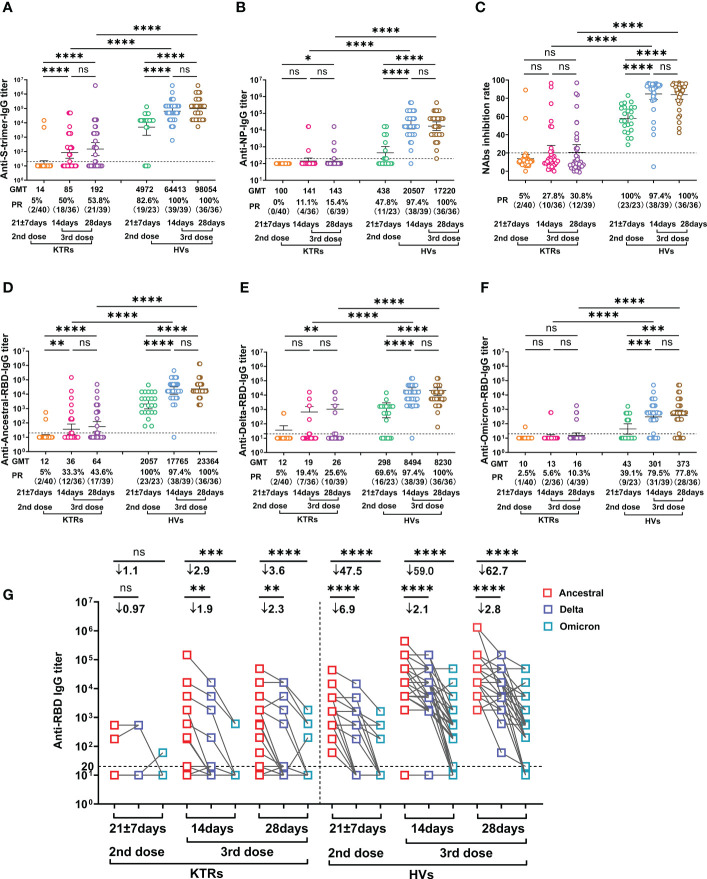
Comparative analysis of SARS-CoV-2 specific antibody IgG between kidney transplant recipients (KTRs) and healthy volunteers (HVs) after the second and third homologous booster. **(A, B)** anti-Ancestral-Spike (S) -trimer-IgG **(A)** and anti-Ancestral nucleocapsid protein (NP)-IgG **(B)** titers in KTRs and HVs 14-28 days after the second vaccination with inactivated vaccine and 14 and 28 days after the third homologous booster. **(C)** Neutralizing antibodies (NAbs) inhibition rate measured by surrogate virus neutralization test in KTRs and HVs 21 ± 7 days post second vaccination with inactivated vaccine and 14 and 28 days after the third homologous booster. **(D-F)** Anti-Ancestral- **(D)**, anti-Delta- **(E)**, anti-Omicron-RBD **(F)** IgG titer in KTRs and HVs 14-28 days after the second vaccination with inactivated vaccine and 14 and 28 days post third homologous booster. **(G)** Paired analysis of Anti-Ancestral-, anti-Delta- and anti-Omicron-RBD IgG titer in KTRs and HVs 14-28 days post second vaccination with inactivated vaccine and 14 and 28 days post third homologous booster. Bar: geometric mean titers (GMT) and 95% confidence interval; dotted line: the detection limits; PR: positive rate. Statistical differences were determined by Mann-Whitney U test. P-values were indicated by ns, not significant (P > 0.05), * (p < 0.05), ** (p < 0.01), *** (p < 0.001), and **** (p < 0.0001).

As expected, the GMT of anti-Ancestral-spike-trimer-IgG was significantly increased in HVs after the third homologous inactivated vaccine booster compared to that at 14-28 days post second dose (**Figure 1A**). Consistently, the GMT of anti-Ancestral-spike-trimer-IgG was also increased in KTRs relative to that at 14-28 days after the second dose, but significantly lower than in HVs after booster (**Figure 1A**). Based on a positive threshold of 1:20, the seroconversion rate of anti-Ancestral-spike-trimer-IgG in KTRs after booster was significantly higher than that after the second dose but markedly lower than that in HVs after booster (**Figure 1A**). Moreover, anti-NP-IgG GMT and positive rate (PR) was significantly increased in HVs after the third booster and slightly increased in KTRs (**Figure 1B**). Then, the NAbs levels were determined using a surrogate virus neutralization test. As shown in **Figure 1C**, the seroconversion of NAbs was observed in 30.8% (12/39) KTRs after the third homologous booster, higher than that after the second dose (5%, 2/40) but markedly lower than that in HVs after the booster (100%) (**Figure 1C**). SARS-CoV-2 variants might escape the neutralizing function of pre-existing NAbs due to the high mutation of its RBD (21). Therefore, the IgG titers against Ancestral-, Delta- and Omicron-RBD was detected in KTRs and HVs. Compared with the second dose, anti-Ancestral-RBD-, anti-Delta-RBD-, and anti-Omicron-RBD-IgG GMT in HVs was significantly improved following the third homologous booster (**Figures 1D–F**). In addition, anti-Delta-RBD-, and anti-Omicron-RBD-IgG PR in HVs was significantly raised after booster compared to that after second dose vaccination (**Figures 1E, F**). However, the GMT of anti-Delta-RBD- and anti-Omicron-RBD-IgG in HVs at 14 days and 28 days post third booster was 2.1-fold and 59-fold, 2.8-fold and 62.7-fold lower than that of the anti-Ancestral-RBD IgG titer during the same period respectively (**Figure 1G**). Regarding KTRs, the GMT and PR of anti-Ancestral-RBD-IgG, anti-Delta-RBD-IgG, but not anti-Omicron-RBD-IgG, was significantly elevated relative to that after the second dose (**Figures 1D–F**). Moreover, anti-Delta-RBD-IgG and anti-Omicron-RBD-IgG GMT in KTRs after 14 days and 28 days post third booster was 2.9-fold and 1.9-fold, and 4.0-fold and 2.5-fold lower than that of anti-Ancestral-RBD-IgG during the same period respectively (**Figure 1G**). Of note, the PR of anti-Ancestral-RBD-IgG, anti-Delta-RBD-IgG and anti-Omicron-RBD-IgG in KTRs was only 43.6% (17/39), 25.6% (10/39) and 10.3% (4/39), respectively, significantly lower than that in HVs (**Figures 1D–F**).

### Comparative analysis of SARS-CoV-2 specific T cells in KTRs and HVs after homologous booster

**Figure 2:**
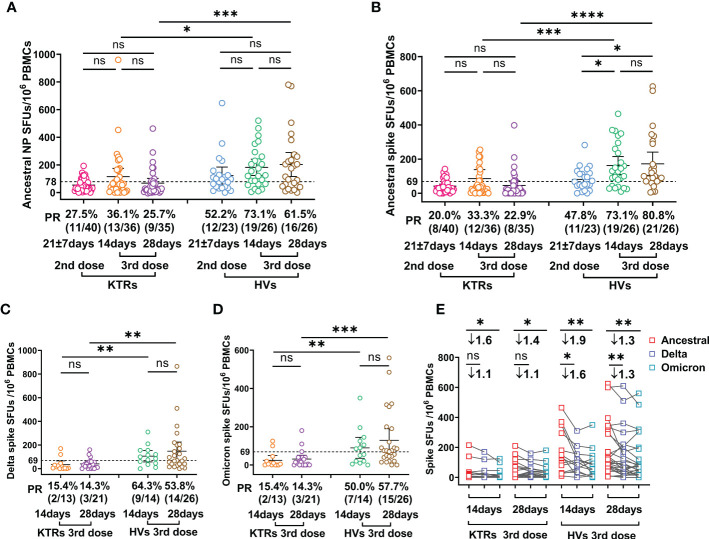
Comparative analysis of SARS-CoV-2 specific T cell response between kidney transplant recipients (KTRs) and healthy volunteers (HVs) after the second booster and third homologous booster. **(A-D)** Ancestral nucleocapsid protein (NP) **(A)**, Ancestral **(B)**, Delta **(C)**, and Omicron **(D)** spike-specific T cell frequency in KTRs and HVs 14-28 days after second vaccination with inactivated vaccine and 14 and 28 days post third homologous booster. **(E)** Paired analysis of Ancestral, Delta and Omicron spike-specific T cell frequency in KTRs and HVs 21 ± 7 days after second vaccination with inactivated vaccine and 14 and 28 days post third homologous booster. Bar: median with interquartile range; dotted line: positive threshold; PBMC: peripheral blood mononuclear cell; SFUs: spot forming units; PR: positive rate. Statistical differences were determined by Mann-Whitney U test. P*-values* were indicated by ns, not significant (P > 0.05), * (p < 0.05), ** (p < 0.01), *** (p < 0.001), and **** (p < 0.0001).

Viral-specific T cells immunity, which mainly functions by clearing virus-infected cells and producing effector cytokines, has critical roles in COVID-19 patient recovery (22, 23). Therefore, we evaluated Ancestral strain spike and NP-, Delta- and Omicron-spike-specific T cells in KTRs and HVs using IFN-γ-based ELISPOTs. The results showed that Ancestral NP-specific T cell frequency was not elevated in KTRs and HVs after the third booster relative to those after the second dose (**Figure 2A**). However, the third booster induced higher Ancestral spike-specific T cell frequency in HVs, but not in KTRs, compared with the second dose (**Figure 2B**). In line with our previous study on the immunogenicity of the second dose (14), the T cells frequency against Ancestral NP and spike in KTRs were significantly lower than that in HVs after the homologous booster, showing an Ancestral spike specific T cells PR of 73.1% (19/26) and 80.8% (21/26) in HVs and 33.3% (12/36) and 22.9% (8/35) in KTRs at 14 and 28 DPB, and an Ancestral NP specific T cells PR of 73.1% (19/26) and 61.5% (16/26) in HVs and 36.1% (13/36)and 25.7% (9/35) in KTRs at 14 and 28 DPB (**Figures 2A, B**). Additionally, KTRs had lower Delta-spike and Omicron-spike-specific T cell frequency than HVs after the third booster (**Figures 2C, D**). Compared with Ancestral spike T cells frequency, Delta-spike and Omicron-spike specific T cells frequency in HVs were 1.6-fold and 1.9-fold lower at 14 DPB and 1.3-fold and 1.3-fold lower at 28 DPB (**Figure 2E**). Moreover, a considerable lower Omicron-spike-specific T cell frequency was observed in KTRs compared with Ancestral spike-specific T cell frequency (**Figure 2E**). However, there was no significant difference between Ancestral-spike- and Delta-spike-specific T cells frequency in KTRs (**Figure 2E**). Based on the previous positive threshold of T cell response against Ancestral spike, 53.8% (14/26) and 57.7% (15/26) of HVs had detectable T cell response against Delta and Omicron spike, and only 14.3% (3/21) of KTRs had detectable T cell immunity against Delta and Omicron spike (**Figures 2C, D**).

### The correlation between SARS-CoV-2 specific cellular and humoral immunity response in KTRs after booster

**Figure 3:**
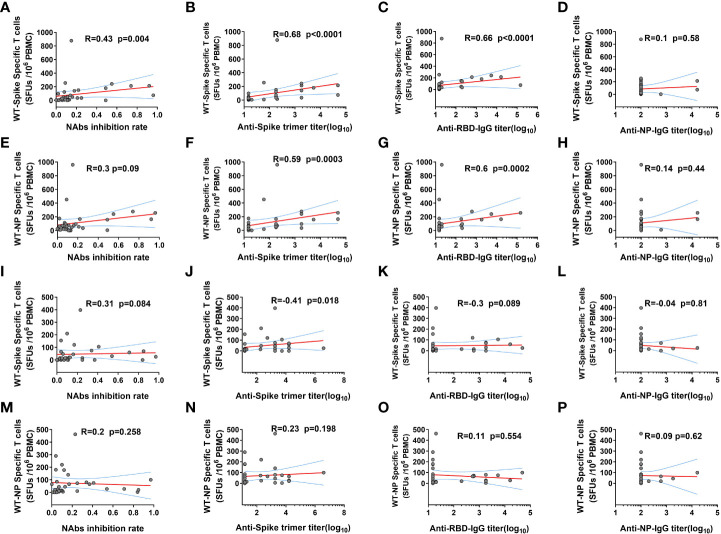
The correlation between SARS-CoV-2 ancestral strain specific T cell and humoral immunity response in kidney transplant recipients (KTRs) after homologous inactivated vaccine booster. **(A-D)** The correlation between SARS-CoV-2 ancestral spike specific T cells frequency and neutralizing antibodies (NAbs) inhibition rate **(A)**, anti-Spike trimer-IgG **(B)**, anti-receptor binding domain (RBD) IgG **(C)**, anti-NP-IgG **(D)** titers 14 days post booster (DPB). **(E-H)** The correlation between SARS-CoV-2 ancestral NP specific T cells frequency and NAbs inhibition rate **(E)**, anti-Spike trimer-IgG **(F)**, anti-RBD-IgG **(G)**, anti-NP-IgG **(H)** titers 14 DPB. The correlation between SARS-CoV-2 ancestral spike specific T cells frequency and NAbs inhibition rate **(I)**, anti-Spike trimer-IgG **(J)**, anti-RBD-IgG **(K)**, anti-NP-IgG **(L)** titers 28 DPB. (M-P) The correlation between SARS-CoV-2 ancestral NP specific T cells frequency and NAbs inhibition rate **(M)**, anti-Spike trimer-IgG **(N)**, anti-RBD-IgG **(O)**, anti-NP-IgG **(P)** titers 28 DPB. SFU: Spot forming units. The correlation was determined using Spearman correlation analysis.

Further correlation analysis demonstrated that ancestral spike specific T cell frequency was positively related to the NAbs level and GMT of anti-Ancestral-spike-trimer-IgG and anti-Ancestral-RBD-IgG, but not anti-Ancestral-NP-IgG at 14 DPB (**Figures 3A–D**), and ancestral NP specific T cell frequency was positively related to GMT of anti-Ancestral-spike-trimer-IgG and anti-Ancestral-RBD-IgG, but not NAbs level and anti-Ancestral-NP-IgG at 14 DPB (**Figures 3E–H**), indicating a coordinated humoral and cellular immunity response in some KTRs at 14 DPB (14). However, the positive correlation was only observed for ancestral spike specific T cell frequency and GMT of anti-Ancestral-spike-trimer-IgG at 28 DPB (**Figures 3I–P**). These dichotomous cellular and humoral immunity response at 28 DPB might be linked to the rapid decline of immunity response in KTRs (24).

### Predictive factors associated with poor SARS-CoV-2 specific NAbs response in KTRs

To dissect the clinical parameters related to the poor SARS-CoV-2 specific NAbs response in KTRs, we further analyzed the difference of pre-boosted clinical laboratory parameters in KTRs who obtained, or did not gain NAbs response (**Table 2**). The results indicated KTRs with positivity of SARS-CoV-2 specific NAbs had higher lymphocyte counts (2.22×10^9^/L versus 1.47×10^9^/L p= 0.003), eosinophils counts (0.10×10^9^/L versus 0.06×10^9^/L, p=0.016), ALT (17.4 U/L versus 14.35 U/L, p=0.039), GGT (37.3 U/L verves 21.45 U/L p=0.022) compared with KTRs without SARS-CoV-2 specific NAbs response (**Table 2**). Multivariate logistic regression analysis was performed using independent variables with p < 0.1 in the univariate analysis. These included female sex, age, lymphocyte counts, eosinophils counts, alanine aminotransferase, gamma glutamyl transpeptidase, hemoglobin, and creatine kinase MB isoenzyme (**Table 2**). This multivariate logistic regression analysis demonstrated that only lymphocyte counts were significantly positively associated with SARS-CoV-2 specific NAbs response in KTRs (OR = 6.483; 95% CI 1.473-28.544, p = 0.013) (**Table 2**).

**Table 2:** Comparative analysis of the pre-boosted demographic characteristics and clinical laboratory parameters between kidney transplant recipients with and without SARS-CoV-2 specific neutralizing antibody response after homologous inactivated vaccine booster.

	KTRs without SARS-CoV-2 neutralizing antibody response (n=27)	KTRs with SARS-CoV-2 neutralizing antibody response (n=12)	*p*-value#	*p*-value*
Female sex number (%)	6(22.2)	2(16.7)	0.692	0.916
Age (year), median (IQR)	41 (15)	44 (13)	0.578	0.435
Time since kidney transplant (months), median (IQR)	42 (51)	78 (59)	0.126	
WBC (×10^9^/L)	7.19(2.89)	7.18(2.58)	0.753	
Neutrophils (×10^9^/L)	4.88(2.74)	4.28(1.85)	0.538	
Lymphocyte (×10^9^/L)	1.47(0.89)	2.22(0.48)	0.003	0.013
Monocyte (×10^9^/L)	0.57(0.30)	0.56(0.32)	0.893	
Eosinophil (×10^9^/L)	0.06(0.03)	0.10(0.11)	0.026	0.193
Basophils(×10^9^/L)	0.03(0.02)	0.03(0.02)	0.313	
RBC (×10^12^/L)	4.58(1.10)	5.18(0.95)	0.461	
Platelet (×10^9^/L)	218 (60)	221.5(101.75)	0.518	
Hemoglobin (g/L)	130 (33)	151(25.75)	0.065	0.629
Tacrolimus (μmol/L)	5.5(2.90)	6.21(1.80)	0.590	
Reticulocyte (×10^9^)	0.076(0.04)	0.078(0.06)	0.940	
IRF (%)	6.90(10.30)	5.85(6.77)	0.620	
LFRR (%)	93.10(10.30)	94.15(6.78)	0.642	
MFRR (%)	6.60(8.60)	5.45(5.65)	0.499	
HFRR (%)	0.40(1.50)	0.15(1.10)	0.374	
C3 (g/L)	1.04(0.23)	1.02(0.24)	0.775	
C4 (g/L)	0.21(0.06)	0.18(0.07)	0.132	
IgG (g/L)	10.0(3.20)	10.75(3.99)	0.149	
IgA (g/L)	2.15 (1.04)	2.16(1.15)	0.775	
IgM (g/L)	0.81(0.64)	0.88(0.96)	0.446	
Cholesterol (mmol/L)	5.5(1.43)	5.27(1.52)	0.776	
Triglyceride (mmol/L)	1.28(0.65)	1.33(0.92)	0.642	
HDL (mmol/L)	1.69(0.50)	1.57(0.89)	0.845	
LDL (mmol/L)	3.21(0.97)	3.10(1.01)	0.869	
Apolipoprotein A (g/L)	1.53(0.57)	1.40(0.47)	0.916	
Apolipoprotein B (g/L)	0.92(0.31)	0.94(0.29)	0.730	
AST (U/L)	19.70(6.32)	22.90(6.80)	0.065	0.089
CK (U/L)	76.50 (40)	90 (45)	0.179	
CK-MB (U/L)	14 (5)	18 (4)	0.052	0.097
LDH (U/L)	172 (68)	178 (54)	0.634	
α-HBDH (U/L)	110.5(47.75)	107 (48)	0.946	
Total Bilirubin (μmol/L)	12.3(5.68)	14.1(8.1)	0.271	
Unconjugated bilirubin (μmol/L)	2.25(1.15)	2.4(1.7)	0.408	
ALT (U/L)	14.35(12.25)	17.4(20.9)	0.039	0.139
ALP (U/L)	49.5(19.25)	66 (44)	0.233	
GGT (U/L)	21.45(18.05)	37.3(40.7)	0.022	0.636
α-L-fucosidase (U/L)	25.85(10.88)	27.3(11.2)	0.558	
Total biliary acid (μmol/L)	3.05(3.87)	2.90(5.80)	0.708	
Urine micro-protein (mg/L)	138 (145)	77(150.75)	0.286	
Urine micro-globulin (mg/L)	0.98(1.06)	8.6(104.38)	0.960	
Urine micro-albumin (mg/L)	29.2(100.9)	0 (0)	0.489	
Urine NAG (U/L)	11 (6)	10(5.75)	0.533	
Urea nitrogen (mmol/L)	6.76(2.46)	7.1(3.09)	0.480	
Creatinine (μmol/L)	110.55(26.80)	105.2(35.4)	0.210	
Albumin (g/L)	43.6(5.12)	44(3.10)	0.443	
Cystatin C(mg/L)	1.20(0.38)	1.09(0.20)	0.270	
Uric acid (μmol/L)	362.5(152.75)	354 (147)	0.391	

KTRs, kidney transplants recipients, WBC, White blood cell, RBC, Red blood cells, IRF, Immature reticulocyte fraction, LFRR, Low fluorescence reticulocyte ratio, MFRR, Median fluorescence reticulocyte ratio, HFRR, High fluorescence reticulocyte ratio, HDL, High-density lipoproteins, LDL, Low density lipoproteins, AST, Aspartate transaminase, CK, Creatine Kinase, LDH, Lactic dehydrogenase, α-HBDH, α-Hydroxybutyrate dehydrogenase, ALT, Alanine Aminotransferase, ALP, Alkaline Phosphatase (U/L), GGT, Gamma Glutamyl Transpeptidase (U/L), NAG, N-acetyl-β-D-glucosaminidase. # p-value of Univariate analysis. *p-value of Multiple logistic regression analyses.

## Discussion

The safety and efficiency of vaccination against the investigated SARS-CoV-2 variants are of greater relevance for KTRs. Herein, we found that the homologous inactivated vaccine booster had no detrimental effects on the kidney, liver and heart function in HVs and KTRs. The humoral immune response against the Ancestral strain was significantly boosted by the homologous inactivated vaccine booster in HVs and KTRs compared to two-dose scheme. Nonetheless, the SARS-CoV-2 specific humoral and cellular immune response in KTRs was significantly lower than in HVs. Moreover, the seroconversion of antibodies against the Ancestral strain and the Delta and Omicron variants RBD was only observed in 43.6% (17/39), 25.6% (10/39) and 10.3% (4/39) KTRs, respectively. In contrast, 100% (36/36), 100% (36/36) and 77.8% (28/36) of HVs had antibodies against the Ancestral strain and the Delta and Omicron variants RBD after homologous inactivated vaccine booster. The T cells immunity response was not improved in KTRs after third booster. Positive T cells against ancestral-NP, ancestral -spike, Delta-spike and Omicron -spike was only observed in 36.1% (13/36), 33.3% (12/36), 14.3% (3/21), and 14.3% (3/21) KTRs.

A previous study reported abnormal kidney, hepatic and heart-related biomarkers and blood cells in COVID-19 patients, implying that injuries to these organs might be the main causes of COVID-19 death (25). Moreover, a recent study demonstrated that two doses of SARS-CoV-2 inactivated vaccine might lead to elevated serum total bile acid, creatinine and cholesterol but decreased potassium levels in healthy individuals (20). However, we did not observe any abnormal hepatic and kidney function-related biomarkers and cholesterol in KTRs and HVs after the third homologous booster. Interestingly, immature reticulocyte percentage was elevated in HVs, but not in KTRs. Immature reticulocytes, a biomarker reflecting erythropoietic activity, are released in the peripheral blood when erythropoiesis is acutely increased above baseline (stress erythropoiesis) (26). However, the reason underlying the increase in immature reticulocytes induced by vaccination is unknown.

SARS-CoV-2 spike-specific antibodies, especially NAbs, are crucial biomarkers for protective humoral immunity against COVID-19 (27). Our previous study indicated that 5% (2/40) of KTRs developed NAbs after two doses of the inactivated vaccine (14). The current study indicated that a third homologous booster significantly increased the seroconversion ratio of NAbs against Ancestral strain in HVs and KTRs. Interestingly, the PR of anti-spiker-trimer IgG against Ancestral strain increased to 53.8% (21/39) in KTRs following homologous booster, higher than that of the NAbs PR (30.8%, 12/39), indicating that the detection target may have a significant impact on immunity response evaluation. However, only 25.6% (10/39) and 10.3% (4/39) of KTRs developed antibodies directed to RBD of Delta and Omicron variants, which are the currently predominant circulating strain (2). Therefore, KTRs without the Delta and Omicron variant-specific antibody still have a higher risk of breakthrough infection from these variants (28, 29). In line with our study, the third mRNA vaccine booster induced a lower number of binding antibodies and NAbs against Omicron variants in KTRs (11, 12). Different from healthy peoples with slower decay rates of neutralization titers over the 6 months after booster-dose administration (30), a rapid decline of both humoral and cellular immunity 6 months after a third mRNA vaccine dose in KTR was observed (24). Altogether, the above data indicate that a fourth dose of the heterologous booster with Omicron variant matched vaccine is urgent need for these low immunogenicity KTRs to improve the immunity against current predominant circulating Omicron variants (31, 32).

Cellular immunity plays a critical role in SARS-CoV-2 clearance and recovery of COVID-19 patients (23). Our previous study showed that inactivated vaccines could induce cellular immunity against SARS-CoV-2 structure protein NP and spike in KTRs, despite being lower than in HVs (14). In contrast to HVs with markedly enhanced SARS-CoV-2 spike-specific T cell response, the SARS-CoV-2 NP and spike-specific T cell immunity were not significantly increased in KTRs after homologous inactivated vaccine booster. Additionally, the PR of SARS-CoV-2 NP and spike-specific T cell immunity in KTRs after homologous booster was not significantly increased compared with that after two doses of inactivated vaccine. These data indicated that homologous inactivated vaccine booster could not further increase the T cell immunity against SARS-CoV-2 in KTRs. Of note, Delta and Omicron variant spike-specific T cell frequency was significantly lower than Ancestral strain spike-specific T cell frequency in HVs and KTRs. The diminished humoral and cellular immunity directed to Omicron variants suggested that these KTRs have higher risk of breakthrough infection, even severe infection (33, 34) and the heterologous mRNA vaccine with higher cellular immunity-induced capacity should be explored in KTRs (13). Recent mice experiment indicated that individuals with lower immunity response elicited by primary immunity might obtain more benefit from an Omicron-matched mRNA vaccine booster than an mRNA-1273mRNA booster (35). In addition, vaccines with multiple conserved T cell epitopes across the whole genome of SARS-CoV-2 targets might benefit low immunogenicity KTRs (23). These results suggest that exploring mRNA vaccine, especially Omicron-matched mRNA vaccine and UB-612 with designed multiple protein/conserve peptide subunit vaccine, might provide important indications to help improve the cellular immunity of KTRs (35, 36).

Few studies have explored the predictive factors related to the humoral immunity response against SARS-CoV-2 in KTRs after vaccination (37). In this study, we found that lymphocyte counts was the only individual factor positively related to humoral immunity response in KTRs. Accordingly, Marion et al. reported solid organ transplant patients, who developed anti-SARS-CoV-2 antibodies had a higher lymphocyte count before vaccination compared to non-seroconverted recipients (38). Further, a study with a large sample of 2092 KTRs demonstrated lymphocyte count appeared important in the prediction of non-seroconversion (37). In addition, almost enrolled KTRs in our study are administered with mycophenolate mofetil (MMF) and prednisone, which was proved as strong detrimental factors of humoral immunity response against SARS-CoV-2 in KTRs (37, 39). MMF preferentially inhibits T and B lymphocytes to suppress cell-mediated immunity response and antibody formation, its common side effect is lymphopenia (38). Therefore, the poor humoral immunity response in our enrolled KTRs might be associated with the different reaction of different patients to MMF.

In summary, a homologous inactivated 3rd booster vaccine might significantly improve the humoral immunity, but not cellular immunity, of KTRs against the Ancestral strain. However, the humoral and cellular immunity of KTRs against the Delta and Omicron variants are still poor despite the homologous inactivated vaccine booster. Novel strategies to improve immunity against Omicron variants in KTRs need to be extensively explored in the future.

## Data availability statement

The original contributions presented in the study are included in the article/**Supplementary Material**. Further inquiries can be directed to the corresponding authors.

## Ethics statement

This observational study was conducted in line with the Declaration of Helsinki and was approved by the Ethics Committee of the Second Affiliated Hospital of Guangzhou Medical University (Approval No. 2021-hs-43). The patients/participants provided their written informed consent to participate in this study.

## Author contributions

Conceptualization: TJ Volunteer recruiting, clinical sample collection, experiments and data analysis: LZ, JY, CL, LW, SX, WK, ZL, PY, MC, WM, MD, LC, YL, and QZ. Manuscript writing and editing: TJ, PL, and SK. Funding acquisition: TJ, ZC, and LZ. Supervision: TJ, PL, ZC, and NY. All authors contributed to data analysis and interpretation and edited the manuscript.

## Supplementary material

Supplementary Figure 1 The temporal course of significantly changed biomarkers in healthy volunteers (HVs) and kidney transplant recipients (KTRs) during the third homologous inactivated vaccine booster. The dynamic of reticulocyte counts **(A)**, immature reticulocyte fraction (IRF) **(B)**, low fluorescence reticulocyte ratio (LFRR) **(C)** and conjugated bilirubin **(D)** in HVs and KTRs at pre-booster, 7 ± 1, 14 ± 2, 32 ± 4 days post homologous inactivated vaccine booster. The line shows the median and interquartile range. Differences between groups were analyzed using the Mann-Whitney U test.
