# Supplementary material for: Immunity against Delta and Omicron variants elicited by homologous inactivated vaccine booster in kidney transplant recipients
Source: Front Immunol. 2023 Jan 9;13:1042784. doi: 10.3389/fimmu.2022.1042784 (PMC9868555; doi:10.3389/fimmu.2022.1042784)
Supplement: Supplementary file 1 [file Table_1.docx]

Supplemental table1. The dynamic of clinical laboratory biomarker in healthy participants after injection of third dose of inactivated vaccine.

| Variables | Pre-3^rd^ | 7±1 days | 14±2 days | 32±4 days | *p*-value | Normal reference range |
| --- | --- | --- | --- | --- | --- | --- |
| WBC (×10^9^/L) | 7.18±2.45 | 6.99±1.92 | 6.70±1.89 | 6.70±1.86 | 0.744 | 3.5-9.5 |
| Neutrophils (×10^9^/L) | 4.21±2.13 | 4.09±1.56 | 3.84±1.49 | 3.88±1.42 | 0.804 | 1.80-6.30 |
| Lymphocyte (×10^9^/L) | 2.30±0.67 | 2.21±0.67 | 2.15±0.62 | 2.18±0.61 | 0.824 | 1.10-3.20 |
| Monocytes (×10^9^/L) | 0.47±0.17 | 0.47±0.15 | 0.49±0.22 | 0.42±0.16 | 0.509 | 0.10-0.60 |
| Eosinophils (×10^9^/L) | 0.16±0.10 | 0.18±0.12 | 0.17±0.11 | 0.18±0.15 | 0.845 | 0.02-0.52 |
| Basophils (×10^9^/L) | 0.04±0.02 | 0.04±0.02 | 0.04±0.03 | 0.04±0.02 | 0.849 | 0-0.06 |
| RBC (×10^12^/L) | 5.04±0.67 | 5.07±0.73 | 5.01±0.60 | 4.97±0.59 | 0.940 | 4.3-5.8 |
| Hemoglobin(g/L) | 147.84±19.96 | 147.34±19.20 | 146.69±16.16 | 147.37±15.33 | 0.995 | 130-175 |
| Platelet (×10^9^/L) | 253.63±72.23 | 248.25±61.75 | 251.47±58.72 | 256.34±65.04 | 0.968 | 125-350 |
| C3 (g/L) | 1.13±0.20 | 1.14±0.20 | 1.14±0.19 | 1.12±0.19 | 0.982 | 0.79-1.52 |
| C4 (g/L) | 0.22±0.08 | 0.22±0.07 | 0.22±0.07 | 0.21±0.07 | 0.997 | 0.09-0.4 |
| IgG (g/L) | 12.05±2.50 | 12.01±2.61 | 11.81±2.23 | 12.09±2.41 | 0.969 | 7.0-15.0 |
| IgA (g/L) | 2.34±0.99 | 2.28±0.93 | 2.28±0.87 | 2.3±50.88 | 0.985 | 0.7-4.0 |
| IgM (g/L) | 1.09±0.49 | 1.09±0.48 | 1.10±0.49 | 1.12±0.45 | 0.996 | 0.4-2.6 |
| Cholesterol (mmol/L) | 5.00±0.99 | 5.10±1.04 | 5.11±1.11 | 5.20±1.06 | 0.903 | 3.15-5.7 |
| Triglyceride (mmol/L) | 2.05±1.99 | 1.98±1.70 | 2.06±1.67 | 1.79±1.43 | 0.918 | 0.5-1.7 |
| [HDL](http://www.baidu.com/link?url=oPQWhy7bSg3vjNAuVZkWYrkiFcc_snfXWEZcpVF7OKeJDNnNGglPrqSXtYjvac50lVHKxMUknZ40__X3NQtSGcf3NRuMqXTutt7RSt4lzq52Weo82KC1_wKY6VK-KUVj) (mmol/L) | 1.34±0.35 | 1.31±0.30 | 1.28±0.34 | 1.37±0.35 | 0.760 | 0.94-1.54 |
| LDL (mmol/L) | 2.88±0.64 | 2.93±0.73 | 2.97±0.70 | 3.09±0.73 | 0.685 | 1.50-3.37 |
| Apolipoprotein A (g/L) | 1.31±0.25 | 1.25±0.19 | 1.32±0.23 | 1.38±0.21 | 0.170 | 1.2-1.8 |
| Apolipoprotein B (g/L) | 0.92±0.26 | 0.95±0.26 | 0.93±0.68 | 0.96±0.27 | 0.935 | 0.6-1.14 |
| [AST](http://www.baidu.com/link?url=TbPLqJNGQDpPW_N7CEgtRRn1WzmyqkUzvS5gt9rVg_MBuRARX0B6J67bbyZFVMxk7vytigEYIjVlg30rRskyu-wVROctOF7-ur612B07BxbGewAeQz0PsSN0obz_hXgx) (U/L) | 25.93±10.03 | 21.84±6.27 | 22.94±8.40 | 25.33±8.75 | 0.181 | 15-40 |
| CK (U/L) | 156.91±185.88 | 122.19±69.66 | 123.88±67.63 | 154.86±149.47 | 0.561 | 50-310 |
| CK-MB (U/L) | 15.84±7.22 | 15.09±7.21 | 12.03±4.43 | 13.24±5.34 | 0.063 | 0-25 |
| LDH (U/L) | 164.03±35.52 | 154.56±22.43 | 147.16±20.29 | 157.86±24.49 | 0.084 | 120-250 |
| α-HBDH (U/L) | 92.97±30.78 | 94.13±26.74 | 76.00±30.15 | 89.21±29.21 | 0.057 | 72-182 |
| [Total Bilirubin](http://www.baidu.com/link?url=q5mAbg2jM8OL_b5t-lzz_bVh_7HIylKad8I6sLvRxlvoilwkjRQx7ZUZyW_pkxvP) (umol/L) | 11.92±5.02 | 11.98±4.91 | 12.88±6.51 | 12.17±4.33 | 0.880 | 6-22 |
| Unconjugated bilirubin (umol/L) | 9.84±4.23 | 10.66±4.59 | 10.81±5.51 | 10.44±3.92 | 0.845 | 1-20 |
| ALT (U/L) | 30.52±21.37 | 25.37±15.16 | 26.21±21.29 | 30.80±19.83 | 0.583 | 9-50 |
| ALP (U/L) | 65.50±23.34 | 66.09±19.32 | 71.13±24.14 | 71.10±24.65 | 0.640 | 45-125 |
| GGT (U/L) | 32.77±25.56 | 32.55±24.45 | 29.89±20.29 | 30.91±24.80 | 0.957 | 10-60 |
| α-L-fucosidase (U/L) | 30.82±7.12 | 29.17±7.10 | 30.56±7.52 | 32.21±8.35 | 0.506 | 14.3-39.9 |
| Total biliary acid (umol/L) | 7.71±13.11 | 5.28±7.76 | 7.58±13.54 | 7.03±9.45 | 0.814 | 0-10 |
| Urine micro-protein (mg/L) | 87.45±58.64 | 62.81±32.48 | 71.94±40.23 | 97.38±85.17 | 0.117 | Unavailable |
| Urine micro-globulin (mg/L) | 0.73±1.00 | 0.89±0.53 | 0.68±0.51 | 0.40±0.31 | 0.042 | 0.1-0.3 |
| Urine micro-albumin (mg/L) | 11.04±14.18 | 7.64±10.86 | 6.66±11.78 | 13.48±34.95 | 0.554 | Unavailable |
| Urine NAG (U/L) | 10.06±4.57 | 8.54±2.45 | 10.34±4.29 | 11.37±7.30 | 0.216 | 0.3-12 |
| Urea nitrogen (mmol/L) | 4.97±0.89 | 4.67±1.03 | 4.66±0.94 | 5.02±1.35 | 0.383 | 2.86-8.2 |
| Creatinine (umol/L) | 86.75±12.75 | 86.94±20.47 | 107.26±135.77 | 83.95±10.46 | 0.526 | 62-115 |
| Albumin (g/L) | 45.69±2.33 | 45.34±2.94 | 44.76±2.79 | 45.21±2.61 | 0.589 | 40-55 |
| Cystatin C(mg/L) | 0.83±0.15 | 0.89±0.13 | 0.83±0.17 | 0.83±0.11 | 0.215 | 0.59-1.03 |
| Uric acid (umol/L) | 422.53±128.86 | 400.31±123.85 | 401.88±128.95 | 405.59±123.67 | 0.887 | 90-420 |
| WBC: White blood cell, RBC: Red blood cells, IRF: Immature reticulocyte fraction, LFRR: Low fluorescence reticulocyte ratio, MFRR: Median fluorescence reticulocyte ratio, HFRR: High fluorescence reticulocyte ratio, HDL: [High-density lipoproteins](http://www.baidu.com/link?url=oPQWhy7bSg3vjNAuVZkWYrkiFcc_snfXWEZcpVF7OKeJDNnNGglPrqSXtYjvac50lVHKxMUknZ40__X3NQtSGcf3NRuMqXTutt7RSt4lzq52Weo82KC1_wKY6VK-KUVj), LDL: Low density lipoproteins, AST: [Aspartate transaminase](http://www.baidu.com/link?url=TbPLqJNGQDpPW_N7CEgtRRn1WzmyqkUzvS5gt9rVg_MBuRARX0B6J67bbyZFVMxk7vytigEYIjVlg30rRskyu-wVROctOF7-ur612B07BxbGewAeQz0PsSN0obz_hXgx), CK: Creatine Kinase, LDH: Lactic dehydrogenase, α-HBDH: α-Hydroxybutyrate dehydrogenase, ALT: Alanine Aminotransferase, ALP: Alkaline Phosphatase (U/L), GGT: Gamma Glutamyl Transpeptidase (U/L), NAG: N-acetyl-β-D-glucosaminidase. | | | | | | |

Supplemental table2. The dynamic of clinical laboratory biomarker in kidney transplant recipients after injection of third dose of inactivated vaccine.

| Variables | Pre-3^rd^ | 7±1 | 14±2 | 32±4 | p-value | Normal reference range |
| --- | --- | --- | --- | --- | --- | --- |
| WBC (×10^9^/L) | 7.31±1.94 | 7.58±2.02 | 7.13±2.42 | 6.99±1.71 | 0.623 | 3.5-9.5 |
| Neutrophils (×10^9^/L) | 4.80±1.63 | 4.91±1.61 | 4.61±2.00 | 4.43±1.29 | 0.626 | 1.80-6.30 |
| Lymphocyte (×10^9^/L) | 1.78±0.59 | 1.95±0.64 | 1.79±0.67 | 1.85±0.67 | 0.632 | 1.10-3.20 |
| Monocytes (×10^9^/L) | 0.62±0.21 | 0.62±0.16 | 0.62±0.25 | 0.58±0.18 | 0.807 | 0.10-0.60 |
| Eosinophils (×10^9^/L) | 0.09±0.06 | 0.08±0.07 | 0.08±0.06 | 0.10±0.07 | 0.759 | 0.02-0.52 |
| Basophils (×10^9^/L) | 0.03±0.02 | 0.03±0.02 | 0.03±0.01 | 0.03±0.02 | 0.697 | 0-0.06 |
| RBC (×10^12^/L) | 4.84±0.78 | 4.89±0.85 | 4.85±0.77 | 4.83±0.73 | 0.988 | 4.3-5.8 |
| Hemoglobin (g/L) | 139.18±23.12 | 139.66±23.40 | 140.86±22.88 | 141.24±20.77 | 0.977 | 130-175 |
| Platelet (×10^9^/L) | 213.82±45.34 | 214.37±61.01 | 205.81±50.84 | 206.21±48.13 | 0.825 | 125-350 |
| Urine micro-protein (mg/L) | **250.92**±475.38 | 224.35±543.67 | 237.00±597.75 | 227.09±440.13 | 0.997 | Unavailable |
| Urine micro-globulin (mg/L) | **2.34**±5.68 | 2.39±4.75 | 1.68±2.95 | 2.92±6.22 | 0.790 | 0.1-0.3 |
| Urine micro-albumin (mg/L) | 105.61±215.68 | 94.68±212.70 | 90.66±207.41 | 89.63±184.11 | 0.987 | Unavailable |
| Urine NAG (U/L) | 10.97±3.42 | 10.17±4.73 | 11.00±5.59 | 10.27±3.93 | 0.823 | 0.3-12 |
| C3 (g/L) | **1.03**±0.17 | 1.29±1.60 | 1.04± | 1.00±0.17 | 0.411 | 0.79-1.52 |
| C4 (g/L) | 0.20±0.05 | 0.21±0.06 | 0.20±0.06 | 0.20±0.05 | 0.870 | 0.09-0.4 |
| IgG (g/L) | 10.21±2.56 | 10.42±2.59 | 10.55±2.39 | 10.69±2.46 | 0.993 | 7.0-15.0 |
| IgA (g/L) | 2.20±0.84 | 2.20±0.81 | 2.15±0.79 | 2.19±0.92 | 0.927 | 0.7-4.0 |
| IgM (g/L) | 0.99±0.50 | 1.02±0.52 | 1.04±0.54 | 1.08±0.63 | 0.927 | 0.4-2.6 |
| Cholesterol (mmol/L) | 5.16±1.05 | 5.27±0.95 | 5.28±0.90 | 5.17±0.95 | 0.923 | 3.15-5.7 |
| Triglyceride (mmol/L) | 1.37±0.73 | 1.36±0.79 | 1.40±0.95 | 1.19±0.73 | 0.655 | 0.5-1.7 |
| [HDL](http://www.baidu.com/link?url=oPQWhy7bSg3vjNAuVZkWYrkiFcc_snfXWEZcpVF7OKeJDNnNGglPrqSXtYjvac50lVHKxMUknZ40__X3NQtSGcf3NRuMqXTutt7RSt4lzq52Weo82KC1_wKY6VK-KUVj) (mmol/L) | 1.58±0.48 | 1.57±0.41 | 1.59±0.44 | 1.61±0.41 | 0.987 | 0.94-1.54 |
| LDL (mmol/L) | 2.98±0.67 | 3.04±0.59 | 2.99±0.55 | 2.99±0.60 | 0.976 | 1.50-3.37 |
| Apolipoprotein A (g/L) | 1.52±0.44 | 1.54±0.38 | 1.53±0.49 | 1.54±0.39 | 0.999 | 1.2-1.8 |
| Apolipoprotein B (g/L) | 0.86±0.21 | 0.89±0.19 | 0.85±0.18 | 0.84±0.19 | 0.743 | 0.6-1.14 |
| [AST](http://www.baidu.com/link?url=TbPLqJNGQDpPW_N7CEgtRRn1WzmyqkUzvS5gt9rVg_MBuRARX0B6J67bbyZFVMxk7vytigEYIjVlg30rRskyu-wVROctOF7-ur612B07BxbGewAeQz0PsSN0obz_hXgx) (U/L) | 23.11±8.89 | 22.28±7.31 | 21.64±7.44 | 20.65±8.44 | 0.614 | 15-40 |
| CK (U/L) | 84.32±33.41 | 82.00±33.92 | 77.89±24.56 | 77.89±36.04 | 0.785 | 50-310 |
| CK-MB (U/L) | 16.42±9.79 | 17.21±13.12 | 16.83±13.02 | 14.64±9.43 | 0.800 | 0-25 |
| LDH (U/L) | 175.82±38.01 | 174.76±43.25 | 164.78±38.14 | 165.76±36.39 | 0.495 | 120-250 |
| α-HBDH (U/L) | 114.00±29.40 | 113.71±30.53 | 108.97±23.91 | 108.82±31.30 | 0.789 | 72-182 |
| [Total Bilirubin](http://www.baidu.com/link?url=q5mAbg2jM8OL_b5t-lzz_bVh_7HIylKad8I6sLvRxlvoilwkjRQx7ZUZyW_pkxvP) (umol/L) | 13.06±4.73 | 13.22±4.88 | 12.66±4.60 | 13.05±6.29 | 0.971 | 6-22 |
| Unconjugated bilirubin (umol/L) | 10.68±3.96 | 11.38±4.18 | 10.93±4.28 | 11.21±5.59 | 0.912 | 1-20 |
| ALT (U/L) | 26.74±37.93 | 21.81±17.49 | 20.03±12.51 | 19.24±12.29 | 0.498 | 9-50 |
| ALP (U/L) | 60.97±21.22 | 62.16±20.80 | 66.75±21.38 | 62.47±22.02 | 0.673 | 45-125 |
| GGT (U/L) | 38.86±40.55 | 36.88±32.90 | 35.72±30.37 | 34.65±28.91 | 0.957 | 10-60 |
| α-L-fucosidase (U/L) | 27.23±7.22 | 28.28±6.86 | 28.18±7.49 | 28.53±9.59 | 0.896 | 14.3-39.9 |
| Total biliary acid (umol/L) | 5.52±10.34 | 5.70±7.32 | 5.13±4.36 | 5.97±8.14 | 0.975 | 0-10 |
| Urea nitrogen (umol/L) | 7.40±2.32 | 7.39±2.01 | 7.21±2.07 | 7.09±2.06 | 0.910 | 2.86-8.2 |
| Creatinine (umol/L) | 113.81±27.89 | 116.63±22.84 | 109.73±23.06 | 112.04±28.91 | 0.702 | 62-115 |
| Albumin (g/L) | 43.53±3.37 | 44.55±2.99 | 44.17±3.28 | 45.07±2.77 | 0.331 | 40-55 |
| Cystatin C (mg/L) | 1.20±0.27 | 1.24±0.30 | 1.18±0.26 | 1.23±0.26 | 0.753 | 0.59-1.03 |
| Uric acid (umol/L) | 370.51±108.04 | 370.21±100.42 | 354.72±82.33 | 336.09±87.81 | 0.379 | 90-420 |
| WBC: White blood cell, RBC: Red blood cells, IRF: Immature reticulocyte fraction, LFRR: Low fluorescence reticulocyte ratio, MFRR: Median fluorescence reticulocyte ratio, HFRR: High fluorescence reticulocyte ratio, HDL: [High-density lipoproteins](http://www.baidu.com/link?url=oPQWhy7bSg3vjNAuVZkWYrkiFcc_snfXWEZcpVF7OKeJDNnNGglPrqSXtYjvac50lVHKxMUknZ40__X3NQtSGcf3NRuMqXTutt7RSt4lzq52Weo82KC1_wKY6VK-KUVj), LDL: Low density lipoproteins, AST: [Aspartate transaminase](http://www.baidu.com/link?url=TbPLqJNGQDpPW_N7CEgtRRn1WzmyqkUzvS5gt9rVg_MBuRARX0B6J67bbyZFVMxk7vytigEYIjVlg30rRskyu-wVROctOF7-ur612B07BxbGewAeQz0PsSN0obz_hXgx), CK: Creatine Kinase, LDH: Lactic dehydrogenase, α-HBDH: α-Hydroxybutyrate dehydrogenase, ALT: Alanine Aminotransferase, ALP: Alkaline Phosphatase (U/L), GGT: Gamma Glutamyl Transpeptidase (U/L), NAG: N-acetyl-β-D-glucosaminidase. | | | | | | |
