# Supplementary figures and images for: Immunity against Delta and Omicron variants elicited by homologous inactivated vaccine booster in kidney transplant recipients
Source: Front Immunol. 2023 Jan 9;13:1042784. doi: 10.3389/fimmu.2022.1042784 (PMC9868555; doi:10.3389/fimmu.2022.1042784)

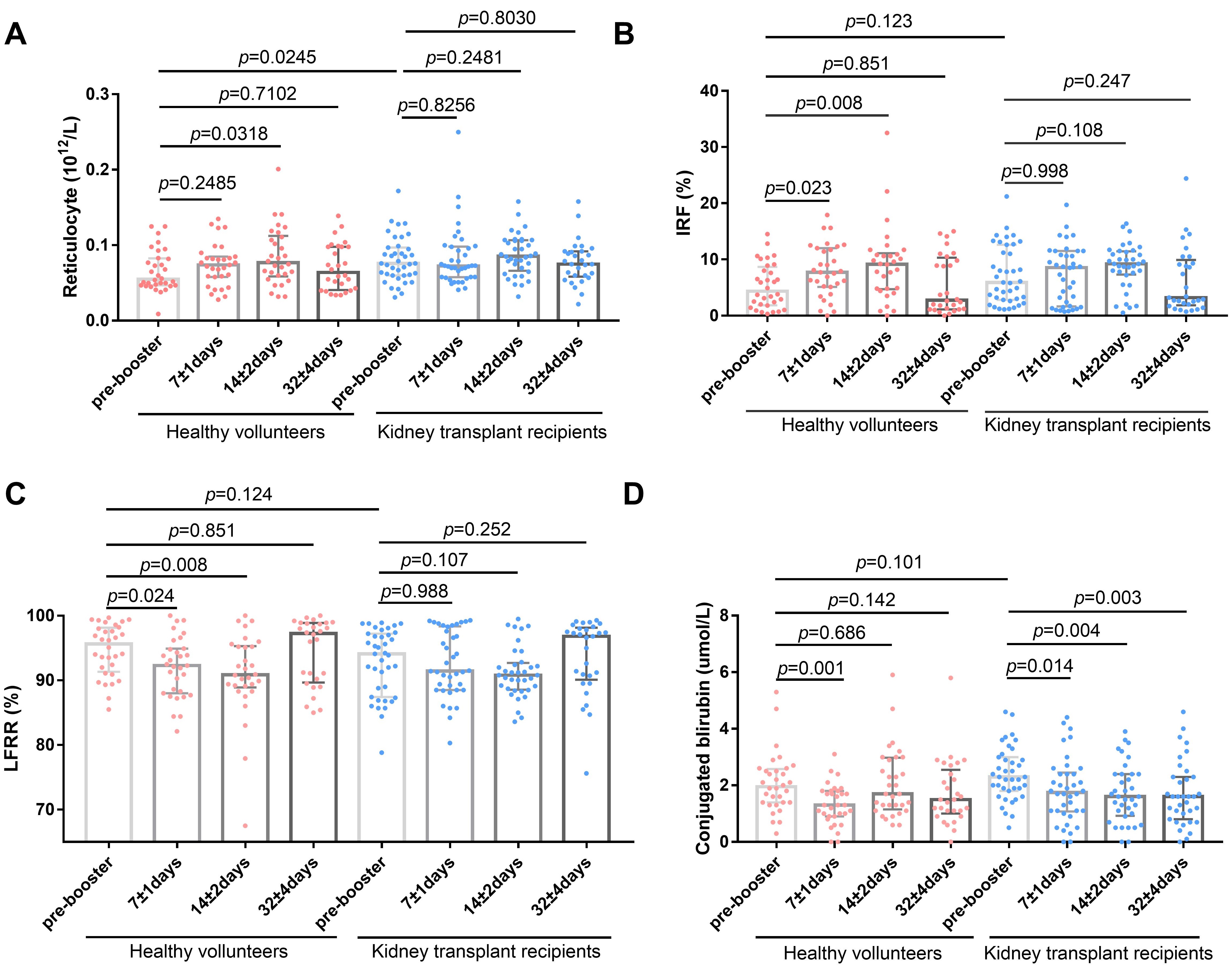

Supplement: Supplementary Figure 1 — The temporal course of significantly changed biomarkers in healthy volunteers (HVs) and kidney transplant recipients (KTRs) during the third homologous inactivated vaccine booster. The dynamic of reticulocyte counts (A), immature reticulocyte fraction (IRF) (B), low fluorescence reticulocyte ratio (LFRR) (C) and conjugated bilirubin (D) in HVs and KTRs at pre-booster, 7 ± 1, 14 ± 2, 32 ± 4 days post homologous inactivated vaccine booster. The line shows the median and interquartile range. Differences between groups were analyzed using the Mann-Whitney U test. [file Image_1.jpeg]
